# Increase of Carbapenem-Resistant *Acinetobacter baumannii* Infection in Acute Care Hospitals in Taiwan: Association with Hospital Antimicrobial Usage

**DOI:** 10.1371/journal.pone.0037788

**Published:** 2012-05-21

**Authors:** Chiu-Hsia Su, Jann-Tay Wang, Chao A. Hsiung, Li-Jung Chien, Cheng-Liang Chi, Hui-Tzu Yu, Feng-Yee Chang, Shan-Chwen Chang

**Affiliations:** 1 Centers for Disease Control (Taiwan), Taipei, Taiwan; 2 Institute of Epidemiology and Preventive Medicine, College of Public Health, National Taiwan University, Taipei, Taiwan; 3 Department of Internal Medicine, National Taiwan University Hospital, Taipei, Taiwan; 4 Institute of Population Health Sciences, National Health Research Institute, Zhunan, Taiwan; 5 Graduate Institute of Pharmacy, College of Medicine, National Taiwan University Hospital, Taipei, Taiwan; East Carolina University School of Medicine, United States of America

## Abstract

**Objective:**

Carbapenem-resistant *Acinetobacter baumannii* (CRAB) has emerged as an important pathogen causing healthcare-associated infections (HAIs) in Taiwan. The present study is aimed to investigate the epidemiology of HAIs caused by CRAB and the association of CRAB infection and hospital usage of different antimicrobials.

**Methods:**

Two nationwide databases in the period 2003 to 2008, the Taiwan Nosocomial Infection Surveillance System and National Health Insurance claim data, were used for analysis. A total of 13,811 healthcare-associated *A. baumannii* infections and antimicrobial usage data from 121 hospitals were analyzed.

**Results:**

There was a significant increase in the proportion of number of HAIs caused by CRAB over that by all *A. baumannii* (CRABpAB), from 14% in 2003 to 46% in 2008 (*P*<0.0001). The greatest increase was in central Taiwan, from 4% in 2003 to 62% in 2008 (*P*<0.0001). Use of anti-pseudomonal carbapenems, but not other classes of antibiotics, was significantly correlated with the increase of CRABpAB (*r* = 0.86, *P*<0.0001).

**Conclusions:**

We suggested that dedicated use of anti-pseudomonal carbapenems would be an important intervention to control the increase of CRABpAB.

## Introduction

*Acinetobacter baumannii* is an important pathogen causing healthcare-associated infections (HAIs) [1] and frequently responsible for outbreaks in healthcare facilities, especially in areas with hot and humid climate [2]. The spread of *A. baumannii* infection is difficult to control because this species can survive for prolonged periods on environmental surfaces. Infection caused by *A. baumannii* is hard to treat since it can acquire resistance to multiple antimicrobial agents [3]. Imipenem and meropenem were traditionally the most effective antimicrobials against *A. baumannii* [4]. However, carbapenem-resistant *A. baumannii* (CRAB) has become common worldwide [5]. In Taiwan, researchers first identified CRAB in the late 1990s, and reported a rapid increase in the late 2000s [6]. The major risk factors for spread of multidrug-resistant organisms (MDROs), including CRAB, are poor adherence to infection control measures and overuse of certain antimicrobials [7].

Previous studies demonstrated that prior use of imipenem and meropenem was an independent risk factor for CRAB infection and/or colonization [8], [9]. However, some researches indicated that emergence of MDROs in a population was dependent on not only the direct effect of individual antimicrobial exposure but also the indirect effect due to increased bacterial resistance in others [10], [11]. This might be because that the higher colonization pressure by MDROs is, the more likely the transmission of MDROs to other patients occurs [12], [13]. Therefore, antimicrobial exposures in other patients might also indirectly affect the risk of acquiring resistant bacteria. Thus, studies that only evaluate individual exposure to antimicrobial agents may under-estimate the total effect of antimicrobial use on the acquisition of resistant bacteria because they do not consider these indirect effects from the antimicrobial exposures in other patients [10]. Ecological studies that use aggregated population-level data may be more suitable for such investigations because they consider the exposure of an entire population.

**Table 1:** Characteristics of the study hospitals and hospital-years according to hospital type, study year, and geographic region.

Demographics	No. (%) of hospitals (n = 121)	No. (%) of hospital-years (n = 409)
Hospital type		
Medical center	16 (13)	74 (18)
Regional hospital	53 (44)	209 (51)
Local hospital	52 (43)	126 (31)
Study year		
2003	-	56 (14)
2004	-	56 (14)
2005	-	66 (16)
2006	-	66 (16)
2007	-	77 (19)
2008	-	88 (22)
Geographic region a		
Taipei	30 (25)	105 (26)
North	18 (15)	62 (15)
Central	29 (24)	78 (19)
South	17 (14)	64 (16)
Kaoping	24 (20)	87 (21)
East	3 (2)	13 (3)

a Taiwan was divided into 6 geographic regions: Taipei, North, Central, South, Kaoping, and East.

The Centers for Disease Control, Taiwan (Taiwan CDC) developed a web-based surveillance system that collects the nationwide HAIs data from intensive care units (ICUs) of participating hospitals and periodically provides feedback to relevant hospitals. Analysis of this collected data indicated a recent overall decrease of HAIs in ICUs of acute care hospitals (medical centers, from 19.8 in 2003 to 12.1 in 2008 per 1000 patient-days; regional hospitals, from 14.1 in 2003 to 9.5 in 2008 per 1000 patient-days) [14], [15]. During this period, *A. baumannii* was the most common causative pathogen of HAI in the ICUs of medical centers (10%) and regional hospitals (12.2%) [14], [15], [16]. Although the overall rate of HAIs decreased, there was an increase in the proportion of number of HAIs caused by CRAB over that by all *A. baumannii* (CRABpAB). In particular, from 2003 to 2008, the CRABpAB increased in the ICUs of medical centers from 16.4% to 56.3% and increased in regional hospitals from 18.2% to 62.1% [14], [15]. It is unknown whether this increase of CRABpAB was associated with the use of certain antimicrobials, but this information is clearly important for the design of interventions to control the increase of CRAB. The aims of the present study are to describe the epidemiological features of HAIs caused by *A. baumannii* and to analyze the association of CRAB infection and hospital usage of different classes of antimicrobial agents.

**Table 2:** Epidemiological characteristics of the 13811 patients with *A. baumannii* infection.

Variables	No. of susceptible isolates (%) (n = 9,496)	No. of resistant isolates (%) (n = 4,315)	OR (95% CI)§
Type of infection			
Bloodstream infection	2,683 (77.3)	786 (22.7)	1.0
Surgical site infection	552 (72.5)	209 (27.5)	1.3 (1.1–1.5)
Pneumonia	3,174 (65.6)	1,667 (34.4)	1.8 (1.6–2.0)
Urinary tract infection	2,126 (64.6)	1,165 (35.4)	1.9 (1.7–2.1)
Other	961 (66.3)	488 (33.7)	1.7 (1.5–2.0)
Hospital type			
Medical center	5,058(71.9)	1,975(28.1)	1.0
Regional hospital	3,403(64.6)	1,866(35.4)	1.4 (1.3–1.5)
Local hospital	1,035(68.6)	474(31.4)	1.2 (1.0–1.3)
Study year			
2003	2,028 (85.9)	334 (14.1)	1.0
2004	1,635 (76.7)	497 (23.3)	1.8 (1.6–2.2)
2005	1,516 (73.7)	540 (26.3)	2.2 (1.9–2.5)
2006	1,300 (70.7)	539 (29.3)	2.5 (2.2–2.9)
2007	1,482 (57.9)	1,079 (42.1)	4.4 (3.8–5.1)
2008	1,535 (53.7)	1,326 (46.3)	5.2 (4.6–6.0)

§ Abbreviations: OR, odds ratio; CI, confidence interval.

**Figure 1:**
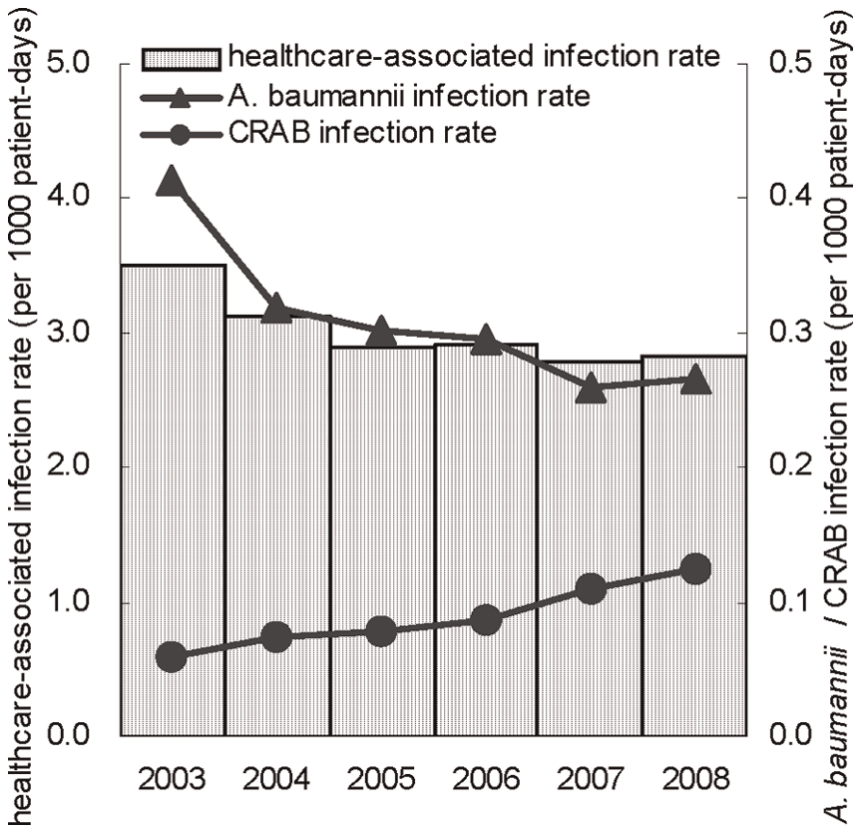
Healthcare-associated infection rate, *A. baumannii* infection rate, and CRAB infection rate from 2003 to 2008.

**Figure 2:**
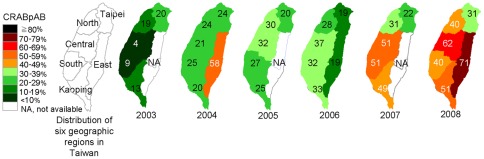
Proportions of CRAB among all *A. baumannii* at hospitals in six geographic regions of Taiwan.

## Methods

### Healthcare-associated *A. baumannii* infections

Data on healthcare-associated *A. baumannii* infections were retrieved from the Taiwan Nosocomial Infections Surveillance system (TNIS) database, which was established in 2002 to collect information about HAIs at participating hospitals. The purposes of the TNIS were to help participating hospitals to develop their own surveillance systems for HAIs and to provide timely recognition of infection control problems. TNIS hospitals voluntarily report demographic data of patients with HAIs, the responsible pathogens for these HAIs, and the antimicrobial susceptibilities (based on the disk diffusion method) of the responsible pathogens. During the study period of 2003 to 2008, a total of 121 hospitals (21 medical centers, 79 regional hospitals, and 21 local hospitals) in Taiwan voluntarily participated in the TNIS. Medical centers in Taiwan generally were referred to hospitals with a capacity of more than 1000 beds and providing tertiary care. Local hospitals were referred to hospitals with a capacity of less than 500 beds and providing primary care. Regional hospitals were those with a size and scale being between the above two types of hospitals. The definition of HAIs during the study period was according to that provided by CDC, USA [17]. Biochemistry methods were used to identify the species of bacterial isolates [18]. Imipenem-resistant or meropenem-resistant *A. baumannii*, as determined by the disk diffusion method described by the Clinical and Laboratory Standards Institute, was considered as CRAB [19]. For every episode of these reported HAIs, only one *A. baumannii* isolate was enrolled for calculation.

**Table 3:** Associations between CRABpAB and hospital antimicrobial usage (DDDs per 1000 patient-days) in 6 regions from 2003 to 2008.

Regions	No. of isolates	Percentage of change of CRABpAB	Percentage of change of antimicrobial usage per 1000 patient-days				
			Anti-pseudomonal cephalosporins	Anti-pseudomonal carbapenems	β-lactam / β-lactamase inhibitor combinations with anti-pseudomonal effect	Anti-pseudomonal fluoroquinolones	Aminoglyco ides
Taipei	2,767	55.6 (19.8–30.8)	15.4 (22.8–26.3)	35.5 (12.4–16.8)	43.8 (11.2–16.1)	18.9 (40.3–47.9)	−53.0 (70.9–33.3)
North	2,896	111.8 (18.7–39.6)	208.5 (9.4–29.0)	84.9 (10.6–19.6)	96.0 (5.0–9.8)	59.2 (28.7–45.7)	−37.9 (83.4–51.8)
Central	2,241	1526.3 (3.8–61.8)	40.2 (12.2–17.1)	215.3 (7.2–22.7)	35.2 (14.5–19.6)	66.4 (22.6–37.6)	–53.4 (98.9–46.1)
South	1,945	361.6 (8.6–39.7)	115.1 (13.9–29.9)	48.4 (12.6–18.7)	142.6 (9.4–22.8)	78.3 (38.3–68.3)	−54.6 (96.2–43.7)
Kaoping	3,818	284.8 (13.2–50.8)	19.6 (19.9–23.8)	103.5 (11.3–23.0)	29.3 (19.8–25.6)	63.2 (27.2–44.4)	−55.9 (111.7–49.3)
East	144	– (NA*–71.2)	46.0 (11.3–16.5)	94.4 (12.4–24.1)	−21.6 (16.2–12.7)	120.8 (32.7–72.2)	−62.1 (98.5–37.3)
Total	13,811	228.4 (14.1–46.3)	50.6 (16.4–24.7)	83.6 (11.0–20.2)	54.5 (12.1–18.7)	49.2 (32.1–47.9)	−51.1 (90.0–44.0)

Percentage of change during the study period was presented from column 3 to 8; and numbers inside parentheses are original data in 2003 and 2008, respectively.

* NA, not available (number of isolates <20 in 2003).

**Figure 3:**
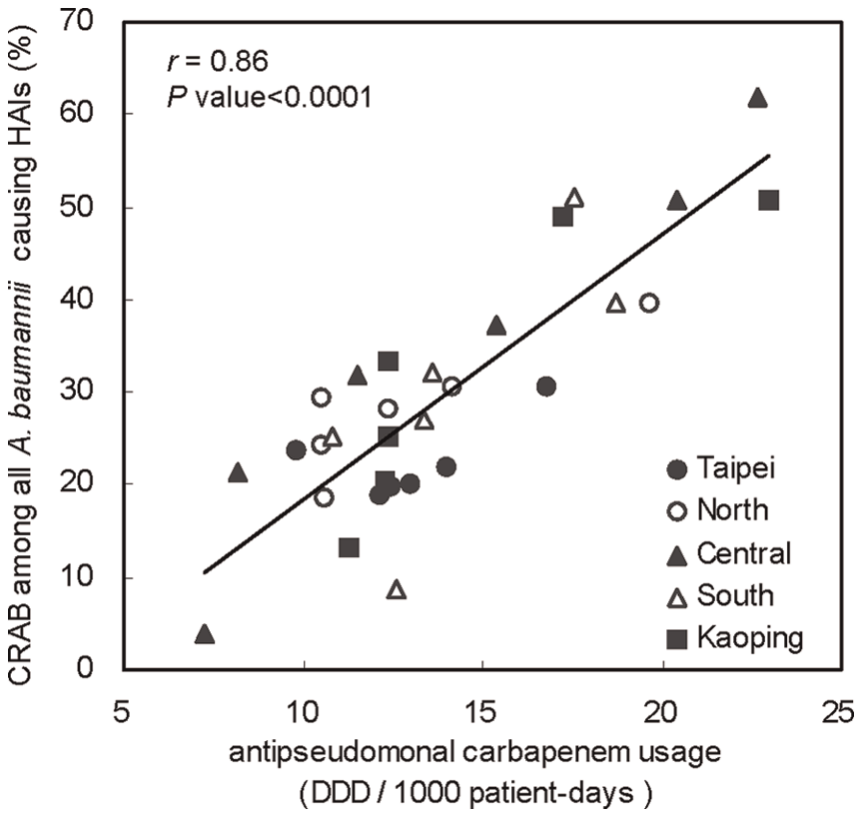
Scattergrams of annual CRABpAB and annual consumption of five classes of antimicrobial agents.

### Hospital antimicrobial usage

Data on inpatient antimicrobial usage were obtained from National Health Insurance (NHI) claim data, which has been collected by the Bureau of National Health Insurance (BNHI) since 1995 for reimbursement purposes. In Taiwan, because of the enforcement of compulsory and universal national health insurance, the coverage rate of NHI is 99% in Taiwanese population and 92% in Taiwanese hospitals [20]. All TNIS hospitals enrolled in this study were covered by NHI. The NHI database contains registration files and original claim data for reimbursement. The BNHI consists of six geographically based branches: Taipei, North, Central, South, Kaoping, and East. The five classes of antimicrobial agents analyzed in this study were: anti-pseudomonal cephalosporins (including cefoperazone, ceftazidime, cefepime, and cefpirome), anti-pseudomonal carbapenems (imipenem/cilastatin, and meropenem), β-lactam-β-lactamase inhibitor combinations with anti-pseudomonal effects (piperacillin/tazobactam, and ticarcillin/clavulanate), anti-pseudomonal fluoroquinolones (ciprofloxacin, and levofloxacin), and aminoglycosides (amikacin, tobramycin, gentamicin, isepamicin, and netilmicin), modified from suggestion by Paterson [9]. The usage of these antimicrobials is transformed as the number of defined daily doses (DDD) per 1000 patient-days, based on the ‘anatomical therapeutical chemical classification/Defined Daily Doses (ATC/DDD) system’ developed by the World Health Organization, [21] and then the usage of antimicrobials belonging to the same antimicrobial group were pooled together. Annual data of antimicrobial use by individual hospitals during the study period (2003–2008) were used.

### Linking data from TNIS and NHI

Each hospital in Taiwan has a unique identification code, and this was used to link hospital data from the TNIS and NHI databases. After this process, the identification codes were fully encrypted to preserve anonymity. To protect the privacy of hospitals, all personal information was kept confidential. This study was conducted according to the Declaration of Helsinki and was approved by the Institutional Review Board (IRB) of Taiwan CDC. The IRB waived the need for informed consents (both written and oral) from participants because this was a retrospective observational study, involved very minimal risk to the subjects, did not include intentional deception, and did not involve sensitive populations or topics; this waiver does not adversely affect the rights and welfare of the subjects.

### Statistical analysis

Data of healthcare-associated *A. baumannii* infections and use of antimicrobial agents were analyzed annually for each of the six regions. Central tendency was expressed as the median and interquartile range (IQR, data between 25^th^ and 75^th^ percentiles). The Chi square test for trend was performed to assess percentages over time and Poisson regression were used for analyzing incidence trends of HAI. The epidemiological features associated with the CRAB infections were assessed by multivariate analysis using a logistic-regression model. Odds ratios and 95 percent confidence intervals (CIs) were calculated for the differences among the characteristics. Correlations (*r*s) between annual CRABpAB and hospital antimicrobial usage were assessed using a repeated-measures mixed-effects model. The East region was excluded in correlation analysis because it had only three hospitals with limited *A. baumannii* isolates, resulting in high variability. *P* values less than 0.05 were considered statistically significant. SAS version 9.2 for Windows (SAS Institute Inc., Cary, NC) was used for all statistical analysis.

## Results

### Demographics of study hospitals

Table 1 shows basic demographic data of the 121 study hospitals. A total of 409 hospital-years were analyzed during the 6-year study period (2003–2008). The median bed capacities of the medical centers, regional hospitals, and local hospitals were 1460, 626, and 183, respectively. The median study duration of participating hospitals was 3 years. The median number of annual admissions was 15287 (IQR: 9368–24607) in 2003 (56 hospitals in that year) and 12289 (IQR: 3920–21132) in 2008 (88 hospitals in that year). The median duration of hospital stays was 10.0 days (IQR: 8.3–11.2) in 2003 and 10.0 days (IQR: 8.4–13.3) in 2008.

### Epidemiological features for CRAB infections

Table 2 shows the basic epidemiological features *A. baumannii* HAIs. A total of 6516 of the 13811 infections (47.2%) occurred in ICUs. The number of HAIs caused by carbapenem-susceptible *A. baumannii* (CSAB) decreased over time, but the number of HAIs caused by CRAB increased over time. Thus, the CRABpAB increased from 14.1% in 2003 to 46.3% in 2008 (*P*<0.0001). Stratification of data by hospital types indicated that the CRABpAB increased from 13.6% in 2003 to 43.9% in 2008 at medical centers (*P*<0.0001), from 15.4% to 49.0% at regional hospitals (*P*<0.0001), and from 13.0% to 44.3% at local hospitals (*P*<0.0001). Regional hospitals had a significantly higher CRABpAB compared to medical centers (odds ratio [OR]: 1.4; 95% confident interval [CI]: 1.3–1.5). After controlling the study years and hospital types, it was found that bloodstream infection was significantly less likely to be caused by CRAB than other infection types.

Figure 1 shows that during the 6-year study period, the average hospital-wide HAI rate of the 121 hospitals decreased from 3.5 per 1000 patient-days in 2003 to 2.8 per 1000 patient-days in 2008 (*P*<0.0001). During this time, the proportion of *A. baumannii* among all pathogens causing HAIs declined from 11.8% in 2003 to 9.4% in 2008 (*P*<0.0001) (data not shown). The infection rate of *A. baumannii* decreased from 0.41 per 1000 patient-days in 2003 to 0.27 per 1000 patient-days in 2008 (*P*<0.0001). However, the infection rate of CRAB doubled during the 6-year period, from 0.06 to 0.12 per 1000 patient-days (*P*<0.0001).

The median duration from admission to *A. baumannii* infection was 23 days (IQR, 13–42) for patients with CRAB infections and 18 days (IQR, 10–33) for those with CSAB infection (*P*<0.0001).

Table 2 shows that the most common CSAB infections were pneumonia (n = 3174, 33%), bloodstream infection (n = 2683, 28%), and urinary tract infection (n = 2126, 22%) and that the most common CRAB infections were pneumonia (n = 1667, 39%), urinary tract infection (n  = 1165, 27%), and bloodstream infection (n = 786, 18%). Among all infections caused by *A. baumannii*, bloodstream infection was significantly less likely to be caused by CRAB than surgical site infection (OR: 1.3; 95% CI: 1.1–1.5), pneumonia (OR: 1.8, 95% CI: 1.6–2.0), urinary tract infection (OR: 1.9; 95% CI: 1.7–2.1), and other infections (OR: 1.7; 95% CI: 1.5–2.0).

Table 2 also shows that there was a significant increase in annual CRABpAB, from 14.1% in 2003 to 46.3% in 2008 (*P*<0.0001). Figure 2 shows geographic differences in CRABpAB (data was not shown for a region if the annual number of infections was less than 20). In 2003, the regions with the highest and lowest CRABpAB were the Taipei region and the central region, respectively. During the six study years, the percentages of *A. baumannii* infection caused by CRAB increased in all six regions. Notable, there was a remarkable increase in the CRABpAB in the central region, from 3.8% in 2003 to 61.8% in 2008, and the central region became the region with highest CRABpAB in 2008. The increase in the Taipei region was more modest, from 19.8% in 2003 to 30.8% in 2008.

### Association of hospital antimicrobial usage and CRABpAB

Table 3 shows the association between the annual CRABpAB and annual hospital antimicrobial usage in the six geographic regions from 2003 to 2008. During the 6-year study period, the annual use of anti-pseudomonal cephalosporins, anti-pseudomonal carbapenems, β-lactam-β-lactamase inhibitor combinations with anti-pseudomonal effect, and anti-pseudomonal fluoroquinolones increased from 16.4 to 24.7, 11.0 to 20.2, 12.1 to 18.7, and 32.1 to 47.9 DDDs per 1000 patient-days, respectively. However, the annual use of aminoglycosides decreased from 90.0 to 44.0 DDDs per 1000 patient-days.

Figure 3 shows scattergrams of annual CRABpAB and annual consumption of each of the five classes antimicrobials stratified by five geographic regions. The results indicate that the annual use of anti-pseudomonal carbapenems was strongly correlated with the increase of annual CRABpAB (*r* = 0.86; *P*<0.0001). None of the other classes of antimicrobial agents was significantly associated with the increase in annual CRABpAB.

## Discussion

Our study of *A. baumannii* HAIs in Taiwan indicated that the CRABpAB increased from 14.1% in 2003 to 46.3% in 2008, and that the incidence rates of HAIs caused by CRAB increased from 0.06 per 1000 patient-days in 2003 to 0.12 per 1000 patient-days in 2008. The increase in CRABpAB was especially remarkable in the central region of Taiwan, which had a 16-fold increase. Our results also indicate that the increase of CRABpAB was strongly associated with the increased use of anti-pseudomonal carbapenems, but not with antimicrobial agents of other classes.

Previous research has reported that the prevalence of carbapenem resistance among *Acinetobacter* species ranged from 6% to 52% in Western countries and 2% to 26% in Asian/Pacific countries other than Taiwan [5]. The results of the present study indicate a higher burden of CRAB in Taiwan than other Asian countries. This is a serious concern, because many prior studies have reported that infection caused by CRAB was associated with greater mortality and morbidity [6]. Only limited antibiotics are available for treatment of CRAB infection [22], [23].Therefore, control of the spread of CRAB is a major medical problem. Previous studies have indicated that risk factors for CRAB acquisition were longer hospital stays, longer ICU stays, invasive procedures, admission to a ward with a high density of patients infected with CRAB, and previous exposure to antibiotics (especially carbapenems) [8], [11], [13], [24], [25], [26], [27], [28], [29], [30]. The findings of the present study are similar. Although many of the factors associated with CRAB infection are not modifiable, prior exposure to carbapenems is a modifiable risk factor. Thus, we suggest that more discretion be used in the administration of carbapenems so as to control the transmission and spread of CRAB.

Previous studies of CRAB typically used individual-level and single center data to investigate the association between antibiotic exposure and acquisition of CRAB, and few studies have investigated multiple centers or used aggregated population-level data [8], [10], [13], [24], [26], [27], [28], [29]. Studies using individual-level data would neglect the indirect, and possibly significant, effects of the antibiotics exposure of people who are near the index person [10], [11]. One population-level study only examined data collected from 152 patients admitted at 11 hospitals [11]. Our present study used nation-wide population-level data of 4315 patients with CRAB infection from 121 hospitals. To our best knowledge, the current study is the largest ecological study to investigate the association between antibiotics exposure and acquisition of CRAB. In addition, our results indicate that the increase of CRABpAB occurred at all types of Taiwanese hospitals.

The major limitation of this study is that we observed a population-level of hospital antimicrobial usage on CRABpAB, but did not examine individual-level antimicrobial exposure data simultaneously. We cannot establish a temporal relationship at patient level. We therefore cannot make a definite causal inference. However, because anti-pseudomonal carbapenems are not effectively against CRAB, the increased consumption of anti-pseudomonal carbapenems is more likely to be the cause of the increase in CRABpAB. Moreover, we did not use molecular typing to identify whether there were epidemics caused by CRAB in the study hospitals or not. If epidemics did exist, some other factors, such as problem of hygiene, would play a role in contributing the increase of CRABpAB. However, the incidence rates of *A. baumannii* HAIs and overall HAIs decreased while CRAB HAIs increased during the study periods (figure 1). We therefore considered that hygiene should not be a major factor resulting in the increase of CRABpAB during our study period. Several studies conducted during this period in Taiwan did demonstrate that intra-hospital outbreaks and inter-hospital spread of CRAB were present; and the CRAB strains responsible for these outbreaks are similar in northern, central, and southern Taiwan [31], [32], [33], [34]. However, there were also some studies demonstrating that even in the situation of outbreaks, prior exposure to imipenem and/or meropenem remained an independently factor for subsequent acquirement of CRAB [8], [35], [36]. In this way, even in the presence of outbreaks episodes, selective pressure from antibiotic exposure would be still one of the important factors leading to the increase of CRABpAB.

In conclusion, our multicenter, population-level study demonstrated a high burden of CRAB and a strong association between increased consumption of anti-pseudomonal carbapenems and increase of CRABpAB in Taiwanese hospitals. We suggest that restriction of anti-pseudomonal carbapenems may help to control the further spread of CRAB.
